# A Cross Sectional Examination of the Relation Between Depression and Frequency of Leisure Time Physical Exercise among the Elderly in Jinan, China

**DOI:** 10.3390/ijerph15092041

**Published:** 2018-09-18

**Authors:** Shukang Wang, Wei Ma, Shu-Mei Wang, Xiangren Yi

**Affiliations:** 1 Department of Biostatistics, School of Public Health, Shandong University, 44, Wenhuaxi Street, Jinan 250012, China; wsk2001@sdu.edu.cn; 2 Department of Epidemiology, School of Public Health, Shandong University, 44, Wenhuaxi Street, Jinan 250012, China; weima@sdu.edu.cn (W.M.); wshm@sdu.edu.cn (S.-M.W.); 3 Department of Sport and Health, the College of Physical Education, Shandong University, 17923, Jingshi Street, Jinan 250061, China

**Keywords:** China, physical exercise, depression, cross-sectional study

## Abstract

Depression has become a major global public health problem. Many studies have shown the positive effects of physical exercise on depression. However, few studies have examined the relationship between frequency of leisure time physical exercise and depression without considering the time and intensity of exercise among middle-aged and elderly people of urban communities in northern China. We conducted a cross-sectional survey that included 1604 participants among urban residents aged 50 years or older in China to evaluate how the frequency of physical exercise was related to depression. Our study showed that the prevalence of depression in the urban community of Jinan is 16.52%. For physical exercise, the odds ratios (ORs) and 95% confidence intervals (CIs) for 1~2 times per week, 3~4 times per week and ≥5 times per week were 1.137 (0.661, 1.953), 0.516 (0.304, 0.875) and 0.548 (0.392, 0.768) respectively, with adjustment for age, gender, marital status, BMI, hypertension, previously diagnosed type 2 diabetes, triglyceride, total cholesterol, soy food intake, milk food intake, vegetable and fruit intake and meat intake. We concluded that physically exercising three times a week is associated with a low prevalence of depression.

## 1. Introduction

Depression, including bipolar depression (BD) and unipolar depression (UD), has become a major global public health problem, which can lead to significant disabilities, lower quality of life and increases in mortality, and both BD and UD share common symptomatic and functional impairments. [1]. Simultaneously, depression is also a risk factor for many major diseases, such as hypertension [2], type 2 diabetes mellitus (T2DM) [3], dementia [4], stroke [5] and cardiovascular disease [6]. By 2015, depression became a primary global burden in terms of disease [7]. In middle-aged and elderly individuals, depression is related to many other non-communicable diseases, and it can aggravate the symptoms of chronic diseases. Diabetic patients with depression are more prone to complications of functional disability [8] compared to diabetic patients without depression. In addition, T2DM patients with depression were at increased risk of being non-adherent to their medications than individuals without depression [9]. Among patients with coronary heart disease, depressive symptoms were also associated with decreased physical exercise, reduced medication adherence and poorer sleep quality [10]. It is certain that depression plays a vital role in the development, progression and prognosis of chronic non-communicable diseases. 

Existing evidence shows that physical exercise is associated with a lower likelihood to manifest depression and its psychosocial impairment. The studies of the relationship between physical exercise and depression can be divided into three categories, including prospective studies, cross-sectional studies, and intervention studies. Prospective studies have shown that physical exercise can reduce the incidence of depression [11,12]. Cross-sectional studies illustrated the coexistence of low physical exercise and high depression [13,14]. In some randomized controlled intervention trials [15,16], physical exercise has proven to be an effective treatment measures for depression, and it has been recommended as a component of depression treatment [17]. In addition, adults who routinely engaged in high levels of physical exercise responded more favorably to depression treatments than adults who engaged in low levels of physical exercise [18].

Although the above studies have shown the positive effects of physical exercise on depression, some studies have also reported the opposite findings for the relationship between physical exercise and depression [19,20]. In addition, previous studies have more integrated the frequency, time and intensity of physical exercise. Older people are not suitable for longer and stronger physical exercise because of their physical condition. To our knowledge, few studies have examined the relationship between frequency of leisure time physical exercise and depression among the middle-aged and elderly people in northern Chinese urban communities. Moreover, physical exercise is an economic, safe and modifiable behavior factor for many diseases, including depression [21,22,23]. Thus, we investigated the prevalence of depression and evaluated how the frequency of physical exercise was related to depression in northern Chinese urban communities, Jinan. The findings from this study may provide a scientific basis for the treatment of depression.

## 2. Materials and Methods

### 2.1. Study Population

We conducted a cross-sectional survey among urban residents aged 50 years or older, who were from several randomly selected neighborhoods located in the six city districts of Jinan, China, between 2011 and 2012. According to the inclusion criteria, individuals aged 50 years or older were included, while they must have lived in the selected communities for more than 6 months in the last year and have the ability to answer the questionnaire. A total of 3277 residents completed the questionnaire in this study, but 1673 individuals were excluded due to the absence of information related to marital status, physical exercise, soy food intake, milk food intake, vegetable and fruit intake, meat intake, height, weight, systolic blood pressure (SBP), diastolic blood pressure (DBP), fasting plasma glucose (FPG), triglyceride (TG), total cholesterol (TC), or information about their current use of medications. A total of 1604 participants was included in the final data analysis. This study was approved by the Ethics Committee of the School of Public Health, Shandong University, and written informed consent was obtained from all participants (Code NO.: 20110301).

### 2.2. Investigation and Measurements

Using a self-designed standardized questionnaire, the specially trained investigators obtained basic information on the respondents, such as age, sex, marriage status (yes or no), current smoking status (yes or no), current alcohol intake (yes or no), the frequency of regular exercise (<1 time per week, 1–2 times per week, 3–4 times per week or ≥ 5 times per week), as well as the frequency of soy food intake (≥1 time per day, 3–6 times per week, 1–2 times per week or <1 time per week), the frequency of milk food intake (≥1 time per day, 3–6 times per week, 1–2 times per week or <1 time per week), the frequency of vegetable and fruit intake (≤1 time per day, 2–3 times per day, 4–5 times per day or ≥5 times per day), meat intake (≥200 g per day, 50–200 g per day, 150– <350 g per week or <150 g per week) and depression (no depression, minor depression, medium depression or major depression). 

After fasting overnight, all participants went through a standard physical examination. First, their height and weight were measured without their shoes, heavy clothing and belts by trained nurses. Body mass index (BMI) was calculated as weight (kg) divided by the squared value of height (m). Systolic blood pressure (SBP) and diastolic blood pressure (DBP) were measured in the sitting position by trained examiners using a mercury sphygmomanometer according to a standard protocol. Measurements were taken three times for each participant, with at least an interval of 5 minutes between each measurement, and the averages of the three readings were chosen as the BP values. Laboratory tests included triglycerides (TG), total cholesterol (TC) and fasting plasma glucose (FPG).

### 2.3. Definitions of Hypertension, Previously Diagnosed Type 2 Diabetes Mellitus (PDM) and Depression

Hypertension was defined by SBP ≥ 140 mmHg, DBP ≥ 90 mmHg or the self-reported current use of antihypertensive medications. Previously diagnosed type 2 diabetes mellitus was defined as T2DM diagnosed by doctors except newly diagnosed diabetes. Using Self-Rating Depression Scale questionnaires (SDS) [24], which is a 20-item self-report measure of the symptoms of depression, a depression score was determined for each subject. The SDS asks each participant to score each item, which contains four levels (never or only a few times = 1, sometimes = 2, often = 3 and almost or all the time = 4). 20 items ranges between 0 and 80. The depression index is equal to the individual’s total score from 20 items divided by 80. The depression indexes of 0–0.49, 0.50–0.59, 0.60–0.69 and 0.70–1.00 represent no depression, minor depression, medium depression and major depression, respectively [25].

### 2.4. Statistical Analysis

Descriptive statistics were calculated for all characteristics according to gender and depression or no depression. The numerical variables were expressed as mean and standard deviation (mean ± s.d.), while the categorical variables were expressed as percentages. Two independent samples *t*-tests were used to compare the distribution of age, BMI, TG and TC. Chi-square tests were used to compare the proportion of participants that were married, smoking, drinking alcohol, hypertensive and PDM. Wilcoxon rank-sum tests were used to determine the differences of the ordinal categorical variables, including regular exercise, soy food intake, milk food intake, vegetable and fruit intake, meat intake and depression. Ordered logistic regression was used to examine the relationship between physical exercise and depression. According to the adjusted different covariates, including age, gender, marital status, BMI, hypertension, PDM, TG, TC, soy food intake, milk food intake, vegetable and fruit intake and meat intake, four ordered logistic regression models were established. All statistical analyses were performed with SAS 9.4 software (SAS Institute Inc., Cary, NC, USA).

## 3. Results

This study included 1604 participants (504 men and 1100 women). The average ages of the men and women were 64.44 and 63.37 years old, respectively. Among all the participants, the proportion of no depression, minor depression, medium depression, and major depression were 83.48%, 13.72%, 2.56% and 0.24%, respectively.

Table 1 shows the characteristics of the male and female subjects and their comparison results. Women reported a significantly higher mean TC, and significantly lower prevalence of marriage, smoking, alcohol drinking, hypertension and PDM. Furthermore, women had a younger mean age. Comparisons of rated variables, such as regular exercise, soy food intake, milk food intake, vegetable and fruit intake, meat intake and depression, men reported significantly higher intake of soy food and meat than women.

Table 2 shows the characteristics of subjects with and without depression and their comparative results. Depression reported a higher prevalence of being female and experiencing PDM, and a significantly lower prevalence of marriage. In addition, according to the comparisons of the ranked variables, including regular exercise, soy food intake, milk food intake, vegetable and fruit intake, meat intake and depression, there was only a statistically significant different in the frequency of regular exercise between the depression group and the non-depression group.

Table 3 shows the relationship between physical exercise and depression in the four models based on the different adjusted variables. In the first model, compared with participants with a frequency of physical exercise that was less than 1 time per week the odds ratios (ORs) and 95% confidence intervals (CIs) were 0.996 (0.585, 1.697), 0.495 (0.293, 0.835) and 0.509 (0.366, 0.709) for participants with frequencies of physical exercise that were 1–2 times per week, 3–4 times per week or ≥5 times per week, respectively, without adjustment for any variables. In the second model, compared with participants with a frequency of physical exercise that was less than 1 time per week the ORs and 95% CIs were 1.044 (0.612, 1.783), 0.505 (0.299, 0.854) and 0.519 (0.372, 0.724) for participants with frequencies of physical exercise that were 1–2 times per week, 3–4 times per week or ≥5 times per week, respectively, with adjustment for age and gender. In the third model, compared with participants with a frequency of physical exercise that was less than 1 time per week the ORs and 95% CIs were 1.125 (0.656, 1.928), 0.531 (0.313, 0.900) and 0.541 (0.387, 0.756) for participants with frequencies of physical exercise that were 1–2 times per week, 3–4 times per week or ≥5 times per week, respectively, with adjustment for age, gender, marital status, BMI, hypertension, PDM, TG and TC. In the fourth model, compared with participants with a frequency of physical exercise that was less than 1 time per week the ORs and 95% CIs were 1.137 (0.661, 1.953), 0.516 (0.304, 0.875) and 0.548 (0.392, 0.768) for participants with frequencies of physical exercise that were 1–2 times per week, 3–4 times per week or ≥ 5 times per week, respectively, with adjustment for age, gender, marital status, BMI, hypertension, PDM, TG, TC, soy food intake, milk food intake, vegetable and fruit intake and meat intake.

**Figure 1:**
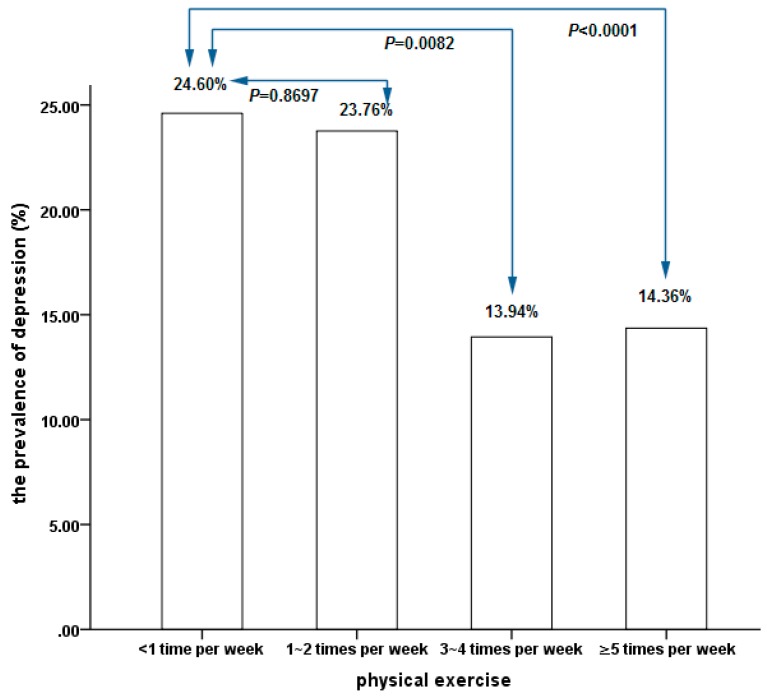
Comparison of the prevalence of depression that was associated with four different frequencies of physical exercise.

Figure 1 shows the comparison of the prevalence of depression that was associated with four different frequencies of physical exercise. Compared with participants with a frequency of physical exercise that was less than 1 time per week there was a significantly lower prevalence of depression in participants with frequencies of physical exercise that were 3–4 times per week or ≥5 times per week. Whereas the difference in the prevalence of depression was not statistically significant between the participants with a frequency of physical exercise that was less than 1 time per week group and 1–2 times per week.

## 4. Discussion

The results of this study show that a frequency of physical exercise of ≥ 3 times every week is associated with a low prevalence of depression. That is, physical exercise of more than three times every week may reduce and improve depression for the elderly without considering the time and intensity of exercise. Some studies, which were not from mainland China, have shown that physical exercise played an important role in the prevention and treatment of depression in older adults [26,27,28,29] and the frequency and duration of physical exercise have always been combined to study the relationship between physical exercise and depression in these studies. However, possibly due to being limited to health conditions [30], older adults rarely adhere to moderate-to-vigorous physical exercise for longer times [31]. Studies have shown that intermittent physical exercise, which does not meet the criteria of each required movement time, also improved the neuromuscular performance [32] and blood pressure [33] for the elderly. Light physical exercise also played an important role in ensuring that the elderly remained healthy [34], and high frequency physical exercise at low intensities may be especially suitable for the elderly [35]. It seems to be reasonable that older adults with high frequencies of physical exercise have more opportunities for community participation. Thus, the results of this study played an important role in the reduction and improvement of depression for the elderly.

With the socio-economic development and changes in lifestyles, the prevalence of depression has increased significantly in China from 3.86% [36] in the late 1990s to about 10–30% [37] currently for the older adults. Although research populations and the measurement tools of depression may be different in different studies, it has been determined that the prevalence of depression has been increasing over the past 30 years in China. A study from western China showed that the prevalence of depression in the urban elderly was as high as 27% [38]. Another study from Changzhi city (Shanxi Province) also reported that the prevalence of depression in the elderly was 26.1% [39]. Our study shows that the prevalence of depression in the urban community of Jinan is 16.52%, similar to the findings for the elderly in Beijing [40]. In China, the population aged 60 years and above accounted for 16.7% of the total population in 2016, so prevention and interventions for depression in the elderly are urgently needed.

Previous epidemiological studies have shown that many factors are associated with depression, including age [41,42], gender [43], obesity [44,45], marital status [46], chronic disease conditions (hypertension [47] and PDM [48]), lifestyle factors (smoking [49], excessive drinking [50], soy food intake [51], vegetable and fruit intake [52], meat intake [53] and physical exercise [54]), and so on. Therefore, in this study, the relationship between the frequency of physical exercise and depression was determined after adjusting for the above factors, and the results show that a frequency of physical exercise that is ≥3 times every week is associated with a low prevalence of depression. Many studies showed that PDM, but not newly diagnosed diabetes (NDM), was significantly associated with depression [55,56]. A study from China also confirmed that patients with PDM demonstrated had increased prevalence of depression, but those individuals with NDM did not [57]. It seems clear that awareness or subsequent treatments of T2DM would negatively impact psychological outcomes [56,58]. Therefore, PDM, not NDM, was adjusted in order to show the influence of physical activity on depression in this study. The biological mechanism between physical exercise and depression remains unclear. According to current studies, physical exercise plays an important role in the prevention and treatment of depression by affecting the structure and function of the brain. For the function of the brain, possible mechanisms include the “endorphin hypothesis” and the “monoamine hypothesis” [59,60,61]. Physical exercise can increase the release of some hormones, such as endorphins [62], norepinephrine [63] and serotonin [64], which can produce happiness in the human body to prevent and treat depression. For the structure of the brain, neuroimaging studies have shown that depression is associated with a decrease in hippocampus volume [65], gray matter [66] or white matter volume [67] in the brain. In addition, UD and BD present both shared and distinctive impairments in the white and grey matter compartments but more white matter abnormalities have been reported in BD than in UD [1]. Physical exercise is associated with larger hippocampus volume [68], white matter and gray matter volumes [69], which may be because physical exercise increases the total length, total volume and total surface area of the capillaries in brain [70].

Depression treatment and prevention are both very important in consideration of the serious situation. Prospective studies, cross-sectional studies and clinical intervention studies are necessary to determine the relationship between physical exercise and depression. Prospective studies are needed to confirm the preventive effects of physical exercise on depression, while determining the role of physical exercise in the treatment of depression simultaneously requires cross-sectional studies and intervention studies. Cross-sectional studies are the basis of interventional trials for depression. We must confirm the coexistence of depression and less frequency physical exercise through cross-sectional studies before implementing clinical intervention trials. Therefore, cross-sectional studies are also important in helping us to find ways of reducing depression.

There are several limitations in the present study. First, although cross-sectional studies are necessary, they can only explain the coexistence of less physical exercise and depression. The causal relationships between the frequency of physical exercise and depression need to be confirmed by intervention experiments and biological mechanisms. Second, about 50% of the residents in this study did not participate in the medical examination, which may lead to bias.

## 5. Conclusions

This study assessed the relationships between the frequency of physical exercise in leisure time and depression among adults aged 50 years and older in Jinan, China. Our results showed that physical exercise was significantly associated with depression with adjustment for age, gender, marital status, BMI, hypertension, PDM, TG, TC, soy food intake, milk food intake, vegetable and fruit intake and meat intake. Furthermore, a frequency of physical exercise that was ≥3 times every week was associated with a low prevalence of depression. Our findings suggest that physical exercise can be one of the interventions for depressed elderly people in urban communities in northern China.

## Figures and Tables

**Table 1:** Summary statistics and comparison of characteristics according to gender (means ± s.d.).

Characteristic	Males (*n* = 504)	Females (*n* = 1100)	Total (*n* = 1604)	*p*
Age (years)	64.44 ± 9.21	63.37 ± 9.67	63.71 ± 9.54	0.0372 *
BMI (kg/m^2^)	25.34 ± 3.35	25.67 ± 8.95	25.56 ± 7.65	0.2865
TG (mg/dL)	1.45 ± 1.01	1.50 ± 0.92	1.49 ± 0.95	0.3408
TC (mg/dL)	4.93 ± 0.85	5.39 ± 1.04	5.25 ± 1.01	<0.0001 **
In marriage, *n* (%)	465 (92.26)	830 (75.45)	1295 (80.74)	<0.0001 **
Smoking, *n* (%)	197 (39.09)	37 (3.36)	234 (14.59)	<0.0001 **
Alcohol drinking, *n* (%)	163 (32.34)	26 (2.36)	189 (11.78)	<0.0001 **
Hypertension, *n* (%)	345 (68.45)	663 (60.27)	1008 (62.84)	0.0016 **
PDM, *n* (%)	77 (15.28)	123 (11.18)	200 (12.47)	0.0212 *
Regular exercise, n (%)				0.3924
<1 time per week	66 (13.10)	186 (16.91)	252 (15.71)	
1–2 times per week	39 (7.74)	62 (5.64)	101 (6.30)	
3–4 times per week	53 (10.52)	112 (10.18)	165 (10.29)	
≥5 times per week	346 (68.64)	740 (67.27)	1086 (67.70)	
Soy food intake, *n* (%)				0.0122 *
≥1 times per day	95 (18.85)	172 (15.64)	267 (16.65)	
3–6 times per week	166 (32.94)	346 (31.45)	512 (31.92)	
1–2 times per week	203 (40.28)	443 (40.27)	646 (40.27)	
Not eating	40 (7.93)	139 (12.64)	179 (11.16)	
Milk food intake, *n* (%)				0.2299
≥1 times per day	173 (34.33)	431 (39.18)	604 (37.66)	
3–6 times per week	71 (14.09)	124 (11.27)	195 (12.16)	
1–2 times per week	77 (15.28)	158 (14.36)	235 (14.65)	
Not drinking	183 (36.30)	387 (35.19)	570 (35.53)	
Vegetable and fruit intake, *n* (%)				0.5127
≤1 times per day	41 (8.13)	76 (6.91)	117 (7.29)	
2–3 times per day	402 (79.76)	886 (80.55)	1288 (80.30)	
4–5 times per day	47 (9.33)	113 (10.27)	160 (9.98)	
≥5 times per day	14 (2.78)	25 (2.27)	39 (2.43)	
Meat intake, *n* (%)				<0.0001 **
≥200 g per day	34 (6.75)	29 (2.64)	63 (3.93)	
50–200 g per day	189 (37.50)	318 (28.91)	507 (31.61)	
150– <350 g per week	173 (34.33)	358 (32.55)	531 (33.10)	
<150 g per week	108 (21.43)	395 (35.91)	503 (31.36)	
Depression, *n* (%)				0.2073
No depression	438 (86.90)	901 (81.91)	1339 (83.48)	
Minor depression	54 (10.71)	132 (15.09)	220 (13.72)	
Medium depression	11(2.18)	30 (2.73)	41 (2.56)	
Major depression	1 (0.21)	3 (0.27)	4 (0.24)	

BMI, body mass index; TG, triglyceride; TC, total cholesterol; PDM, previously diagnosed type 2 diabetes mellitus. *: *p* < 0.05; **: *p* < 0.01.

**Table 2:** Summary statistics and comparison of characteristics according to depression (means ± s.d.).

Characteristic	Depression (*n* = 265)	No depression (*n* = 1339)	*p*
Age(years)	64.66 ± 10.17	63.52 ± 9.40	0.0768
BMI (kg/m^2^)	25.22 ± 3.64	25.63 ± 8.21	0.1938
TG (mg/dL)	1.57 ± 1.07	1.47 ± 0.92	0.1470
TC (mg/dL)	5.27 ± 1.23	5.24 ± 0.95	0.7448
Female, *n* (%)	199 (75.09)	901 (67.29)	0.0124 *
In marriage, *n* (%)	196 (73.96)	1099 (82.08)	0.0022 **
Smoking, *n* (%)	40 (15.09)	194 (14.49)	0.8347
Alcohol drinking, *n* (%)	32 (12.08)	157 (11.73)	0.8716
Hypertension, *n* (%)	176 (66.42)	832 (62.14)	0.1878
PDM, *n* (%)	44 (16.60)	156 (11.65)	0.0257 *
Regular exercise, *n* (%)			0.0001 **
<1 time per week	62 (23.40)	190 (14.19)	
1–2 times per week	24 (9.06)	77 (5.75)	
3–4 times per week	23 (8.68)	142 (10.60)	
≥5 times per week	156 (58.87)	930 (69.45)	
Soy food intake, *n* (%)			0.3066
≥1 times per day	27 (10.19)	240 (17.92)	
times per week	102 (38.49)	410 (30.62)	
1–2 times per week	106 (40.00)	540 (40.33)	
Not eating	30 (11.32)	149 (11.13)	
Milk food intake, *n* (%)			0.2992
≥1 times per day	91 (34.34)	513 (38.31)	
3–6 times per week	33 (12.45)	162 (12.10)	
1–2 times per week	43 (16.23)	192 (14.34)	
Not drinking	98 (36.98)	472 (35.25)	
Vegetable and fruit intake, *n* (%)			0.2724
≤1 times per day	27 (10.19)	90 (6.72)	
2–3 times per day	205 (77.36)	1083 (80.88)	
4–5 times per day	26 (9.81)	134 (10.01)	
≥5 times per day	7 (2.64)	32 (2.39)	
Meat intake, *n* (%)			0.6838
≥200g per day	9 (3.40)	54 (4.03)	
50–200 g per day	85 (32.08)	422 (31.52)	
150– <350 g per week	94 (35.47)	437 (32.64)	
<150 g per week	77 (29.06)	426 (31.81)	

BMI, body mass index; TG, triglyceride; TC, total cholesterol; PDM, previously diagnosed type 2 diabetes mellitus. *: *p* < 0.05; **: *p* < 0.01.

**Table 3:** Odd ratios (ORs) and their 95% confidence intervals (CI) of the risk of physical exercise for depression from ordered logistic model.

Variables	Model^1^	Model^2^	Model^3^	Model^4^
Regular exercise				
<1 time per week	ref	ref	ref	ref
1–2 times per week	0.996 (0.585, 1.697)	1.044 (0.612, 1.783)	1.125 (0.656, 1.928)	1.137 (0.661, 1.953)
3–4 times per week	0.495 (0.293, 0.835)	0.505 (0.299, 0.854)	0.531 (0.313, 0.900)	0.516 (0.304, 0.875)
≥5 times per week	0.509 (0.366, 0.709)	0.519 (0.372, 0.724)	0.541 (0.387, 0.756)	0.548 (0.392, 0.768)
Gender				
male		ref	ref	ref
female		1.447 (1.069, 1.957)	1.381 (1.013, 1.883)	1.382 (1.011, 1.888)
Marital status				
Married			ref	ref
Not married			1.426 (1.038, 1.959)	1.411 (1.024, 1.945)
PDM				
no			ref	ref
yes			1.478 (1.020, 2.142)	1.511 (1.040, 2.197)
Soy food intake, *n* (%)				
≥1 times per day				ref
3–6 times per week				2.187 (1.383, 3.459)
1–2 times per week				1.689 (1.072, 2.661)
Not eating				1.570 (0.889, 2.772)

Model^1^: unadjusted. Model^2^: adjusted by age and gender. Model^3^: adjusted by age, gender, marital status, BMI, hypertension, PDM, TG and TC. Model^4^: adjusted by age, gender, marital status, BMI, hypertension, PDM, TG, TC, soy food intake, milk food intake, vegetable and fruit intake and meat intake. Ref: reference.

## References

[1] Serafini G., Pompili M., Borgwardt S., Houenou J., Geoffroy P.A., Jardri R., Girardi P., Amore M. (2014). Brain changes in early-onset bipolar and unipolar depressive disorders: A systematic review in children and adolescents. Eur. Child Adolesc. Psychiatry.

[2] Meng L., Chen D., Yang Y., Zheng Y., Hui R. (2012). Depression increases the risk of hypertension incidence: A meta-analysis of prospective cohort studies. J. Hypertens..

[3] Vancampfort D., Correll C.U., Galling B., Probst M., De Hert M., Ward P.B., Rosenbaum S., Gaughran F., Lally J., Stubbs B. (2016). Diabetes mellitus in people with schizophrenia, bipolar disorder and major depressive disorder: A systematic review and large scale meta-analysis. World Psychiatry.

[4] Almeida O.P., Hankey G.J., Yeap B.B., Golledge J., Flicker L. (2017). Depression as a modifiable factor to decrease the risk of dementia. Transl. Psychiatry.

[5] Brunner E.J., Shipley M.J., Britton A.R., Stansfeld S.A., Heuschmann P.U., Rudd A.G., Wolfe C.D., Singh-Manoux A., Kivimaki M. (2014). Depressive disorder, coronary heart disease, and stroke: Dose-response and reverse causation effects in the Whitehall II cohort study. Eur. J. Prev. Cardiol..

[6] Pan A., Sun Q., Okereke O.I., Rexrode K.M., Hu F.B. (2011). Depression and risk of stroke morbidity and mortality: A meta-analysis and systematic review. JAMA.

[7] Vos T., Allen C., Arora M., Barber R.M., Bhutta Z.A., Brown A., Carter A., Casey D.C., Charlson F.J., Chen A.Z. (2016). Global, regional, and national incidence, prevalence, and years lived with disability for 310 diseases and injuries, 1990–2015: A systematic analysis for the Global Burden of Disease Study 2015. Lancet.

[8] Kalyani R.R., Ji N., Carnethon M., Bertoni A.G., Selvin E., Gregg E.W., Sims M., Golden S.H. (2017). Diabetes, depressive symptoms, and functional disability in African Americans: The Jackson Heart Study. J. Diabetes Complicat..

[9] Lunghi C., Zongo A., Moisan J., Grégoire J.P., Guénette L. (2017). Factors associated with antidiabetic medication non-adherence in patients with incident comorbid depression. J. Diabetes Complicat..

[10] Sin N.L., Kumar A.D., Gehi A.K., Whooley M.A. (2016). Direction of Association Between Depressive Symptoms and Lifestyle Behaviors in Patients with Coronary Heart Disease: The Heart and Soul Study. Ann. Behav. Med..

[11] Kaseva K., Rosenström T., Hintsa T., Pulkki-Råback L., Tammelin T., Lipsanen J., Yang X., Hintsanen M., Hakulinen C., Pahkala K. (2016). Trajectories of Physical Activity Predict the Onset of Depressive Symptoms but Not Their Progression: A Prospective Cohort Study. J. Sports Med..

[12] Anagnostopoulos A., Ledergerber B., Jaccard R., Shaw S.A., Stoeckle M., Bernasconi E., Barth J., Calmy A., Berney A., Jenewein J. (2015). Swiss HIV Cohort Study. Frequency of and Risk Factors for Depression among Participants in the Swiss HIV Cohort Study (SHCS). PLoS ONE.

[13] Yu C.Q., Chen Y.P., Lv J., Guo Y., Sherliker P., Bian Z., Zhou H.Y., Tan Y.L., Chen J.S., Chen Z.M. (2016). Major depressive disorder in relation with coronary heart disease and stroke in Chinese adults aged 30-79 years. Beijing Da Xue Xue Bao.

[14] Teychenne M., Ball K., Salmon J. (2008). Physical activity and likelihood of depression in adults: A review. Prev. Med..

[15] Ranjbar E., Memari A.H., Hafizi S., Shayestehfar M., Mirfazeli F.S., Eshghi M.A. (2015). Depression and Exercise: A Clinical Review and Management Guideline. Asian J. Sports Med..

[16] Lok N., Lok S., Canbaz M. (2017). The effect of physical activity on depressive symptoms and quality of life among elderly nursing home residents: Randomized controlled trial. Arch. Gerontol. Geriatr..

[17] Ledochowski L., Stark R., Ruedl G., Kopp M. (2017). Physical activity as therapeutic intervention for depression. Nervenarzt.

[18] Hallgren M., Nakitanda O.A., Ekblom Ö., Herring M.P., Owen N., Dunstan D., Helgadottir B., Forsell Y. (2016). Habitual physical activity levels predict treatment outcomes in depressed adults: A prospective cohort study. Prev. Med..

[19] Cooper-Patrick L., Ford D.E., Mead L.A., Chang P.P., Klag M.J. (1997). Exercise and depression in midlife: A prospective study. Am. J. Public Health.

[20] Kritz-Silverstein D., Barrett-Connor E., Corbeau C. (2001). Cross-sectional and prospective study of exercise and depressed mood in the elderly: The Rancho Bernardo study. Am. J. Epidemiol..

[21] Memari A.H., Ghanouni P., Shayestehfar M., Ziaee V., Moshayedi P. (2014). Effects of visual search vs. auditory tasks on postural control in children with autism spectrum disorder. Gait Posture.

[22] Memari A.H., Ghaheri B., Ziaee V., Kordi R., Hafizi S., Moshayedi P. (2013). Physical activity in children and adolescents with autism assessed by triaxial accelerometry. Pediatr. Obes..

[23] Memari A.H., Ziaee V., Beygi S., Moshayedi P., Mirfazeli F.S. (2012). Overuse of psychotropic medications among children and adolescents with autism spectrum disorders: Perspective from a developing country. Res. Dev. Disabil..

[24] Zung W.W. (1965). A self-rating depression scale. Arch. Gen. Psychiatry.

[25] Wang X.D., Jiang C.Q., Ma H. (1999). Rating Scales for Mental Health.

[26] Bridle C., Spanjers K., Patel S., Atherton N.M., Lamb S.E. (2012). Effect of exercise on depression severity in older people: Systematic review and meta-analysis of randomised controlled trials. Br. J. Psychiatry.

[27] Schuch F.B., Vancampfort D., Richards J., Rosenbaum S., Ward P.B., Stubbs B. (2016). Exercise as a treatment for depression: A meta-analysis adjusting for publication bias. J. Psychiatr. Res..

[28] Schuch F.B., Vancampfort D., Rosenbaum S., Richards J., Ward P.B., Veronese N., Solmi M., Cadore E.L., Stubbs B. (2016). Exercise for depression in older adults: A meta-analysis of randomized controlled trials adjusting for publication bias. Rev. Bras. Psiquiatr..

[29] Mammen G., Faulkner G. (2013). Physical activity and the prevention of depression: A systematic review of prospective studies. Am. J. Prev. Med..

[30] Schutzer K.A., Graves B.S. (2004). Barriers and motivations to exercise in older adults. Prev. Med..

[31] Aoyagi Y., Shephard R.J. (2010). Habitual physical activity and health in the elderly: The Nakanojo Study. Geriatr. Gerontol. Int..

[32] Thomas E.E., De Vito G., Macaluso A. (2007). Speed training with body weight unloading improves walking energy cost and maximal speed in 75- to 85-yearold healthy women. J. Appl. Physiol..

[33] Nemoto K., Gen-no H., Masuki S., Okazaki K., Nose H. (2007). Effects of high-intensity interval walking training on physical fitness and blood pressure in middle-aged and older people. Mayo Clin. Proc..

[34] Buman M.P., Hekler E.B., Haskell W.L., Pruitt L., Conway T.L., Cain K.L., Sallis J.F., Saelens B.E., Frank L.D., King A.C. (2010). Objective light-intensity physical activity associations with rated health in older adults. Am. J. Epidemiol..

[35] Chang Y.C., Lu M.C., Hu I.H., Wu W.I., Hu S.C. (2017). Effects of different amounts of exercise on preventing depressive symptoms in community-dwelling older adults: A prospective cohort study in Taiwan. BMJ. Open.

[36] Chen R., Copeland J.R., Wei L. (1999). A meta-analysis of epidemiological studies in depression of older people in the People’s Republic of China. Int. J. Geriatr. Psychiatry.

[37] Guo J., Liu C., Wang X., Qu Z., Zhang W., Zhang X. (2017). Relationships between depression, pain and sleep quality with doctor visits among community-based adults in north-west China. Public Health.

[38] Li Y., Chen C., Tu H., Cao W., Fan S., Ma Y., Xu Y., Hua Q. (2012). Prevalence and risk factors for depression in older people in Xi’an China: A community-based study. Int. J. Geriatr. Psychiatry.

[39] Cao W., Guo C., Ping W., Tan Z., Guo Y., Zheng J. (2016). A Community-Based Study of Quality of Life and Depression among Older Adults. Int. J. Environ. Res. Public Health.

[40] Li N., Pang L., Chen G., Song X., Zhang J., Zheng X. (2011). Risk factors for depression in older adults in Beijing. Can. J. Psychiatry.

[41] Kleinberg A., Aluoja A., Vasar V. (2010). Point prevalence of major depression in Estonia. Results from the 2006 Estonian Health Survey. Eur. Psychiatry.

[42] Kolchakova P.Y., Akabaliev V.H. (2003). A study of the effect of age on depressivity in Bulgarian urban population. Folia. Med..

[43] Silva M.T., Galvao T.F., Martins S.S., Pereira M.G. (2014). Prevalence of depression morbidity among Brazilian adults: A systematic review and meta-analysis. Rev. Bras. Psiquiatr..

[44] Jung S.J., Woo H.T., Cho S., Park K., Jeong S., Lee Y.J., Kang D., Shin A. (2017). Association between body size, weight change and depression: Systemic review and meta-analysis. Br. J. Psychiatry.

[45] Qian J., Li N., Ren X. (2017). Obesity and depressive symptoms among Chinese people aged 45 and over. Sci. Rep..

[46] Aluoja A., Leinsalu M., Shlik J., Vasar V., Luuk K. (2004). Symptoms of depression in the Estonian population: Prevalence, sociodemographic correlates and social adjustment. J. Affect. Disord..

[47] Maatouk I., Herzog W., Böhlen F., Quinzler R., Löwe B., Saum KU., Brenner H., Wild B. (2016). Association of hypertension with depression and generalized anxiety symptoms in a large population-based sample of older adults. J. Hypertens..

[48] Hsu Y.M., Su L.T., Chang H.M., Sung F.C., Lyu S.Y., Chen P.C. (2012). Diabetes mellitus and risk of subsequent depression: A longitudinal study. Int. J. Nurs. Stud..

[49] Luger T.M., Suls J., Vander Weg M.W. (2014). How robust is the association between smoking and depression in adults? A meta-analysis using linear mixed-effects models. Addict. Behav..

[50] Graham K., Massak A., Demers A., Rehm J. (2007). Does the association between alcohol consumption and depression depend on how they are measured?. Alcohol Clin. Exp. Res..

[51] Zhou X., Bi B., Zheng L., Li Z., Yang H., Song H., Sun Y. (2014). The prevalence and risk factors for depression symptoms in a rural Chinese sample population. PLoS ONE.

[52] McMartin S.E., Jacka F.N., Colman I. (2013). The association between fruit and vegetable consumption and mental health disorders: Evidence from five waves of a national survey of Canadians. Prev. Med..

[53] Meyer B.J., Kolanu N., Griffiths D.A., Grounds B., Howe P.R., Kreis I.A. (2013). Food groups and fatty acids associated with self-reported depression: An analysis from the Australian National Nutrition and Health Surveys. Nutrition.

[54] Roh H.W., Hong C.H., Lee Y., Oh B.H., Lee K.S., Chang K.J., Kang D.R., Kim J., Lee S., Back J.H. (2015). Participation in Physical, Social, and Religious Activity and Risk of Depression in the Elderly: A Community-Based Three-Year Longitudinal Study in Korea. PLoS ONE.

[55] Bouwman V., Adriaanse M.C., Van’t Riet E., Snoek F.J., Dekker J.M., Nijpels G. (2010). Depression, anxiety and glucose metabolism in the general Dutch population: The new Hoorn study. PLoS ONE.

[56] Nouwen A., Nefs G., Caramlau I., Connock M., Winkley K., Lloyd C.E., Peyrot M., Pouwer F., European Depression in Diabetes Research Consortium (2011). Prevalence of depression in individuals with impaired glucose metabolism or undiagnosed diabetes: A systematic review and meta-analysis of the European Depression in Diabetes (EDID) Research Consortium. Diabetes Care.

[57] Sun J.C., Xu M., Lu J.L., Bi Y.F., Mu Y.M., Zhao J.J., Liu C., Chen L.L., Shi L.X., Li Q. (2015). Associations of depression with impaired glucose regulation, newly diagnosed diabetes and previously diagnosed diabetes in Chinese adults. Diabet. Med..

[58] De G.M., Anderson R., Freedland K.E., Clouse R.E., Lustman P.J. (2001). Association of depression and diabetes complications: A meta-analysis. Psychosom. Med..

[59] Schildkraut J.J. (1973). Neuropharmacology of the affective disorders. Annu. Rev. Pharmacol..

[60] Schildkraut J.J. (1995). The catecholamine hypothesis of affective disorders: A review of supporting evidence. 1965. J. Neuropsychiatry Clin. Neurosci..

[61] Vawter M.P., Freed W.J., Kleinman J.E. (2000). Neuropathology of bipolar disorder. Biol. Psychiatry.

[62] Sinaei M., Kargarfard M. (2015). The evaluation of BMI and serum beta-endorphin levels: The study of acute exercise intervention. J. Sports Med. Phys. Fit..

[63] Daniele T.M.D.C., de Bruin P.F.C., Rios E.R.V., de Bruin V.M.S. (2017). Effects of exercise on depressive behavior and striatal levels of norepinephrine, serotonin and their metabolites in sleep-deprived mice. Behav. Brain Res..

[64] Post R.M., Kotin J., Goodwin F.K., Gordon E.K. (1973). Psychomotor activity and cerebrospinal fluid amine metabolites in affective illness. Am. J. Psychiatry.

[65] Taylor W.D., McQuoid D.R., Payne M.E., Zannas A.S., MacFall J.R., Steffens D.C. (2014). Hippocampus atrophy and the longitudinal course of late-life depression. Am. J. Geriatr. Psychiatry.

[66] Shad M.U., Muddasani S., Rao U. (2012). Gray matter differences between healthy and depressed adolescents: A voxel-based morphometry study. J. Child Adolesc. Psychopharmacol..

[67] Charlton R.A., Lamar M., Zhang A., Yang S., Ajilore O., Kumar A. (2014). White-matter tract integrity in late-life depression: Associations with severity and cognition. Psychol. Med..

[68] Erickson K.I., Leckie R.L., Weinstein A.M. (2014). Physical activity, fitness, and gray matter volume. Neurobiol. Aging.

[69] Gow A.J., Bastin M.E., Muñoz Maniega S., Valdés Hernández M.C., Morris Z., Murray C., Royle N.A., Starr J.M., Deary I.J., Wardlaw J.M. (2012). Neuroprotective lifestyles and the aging brain: Activity, atrophy, and white matter integrity. Neurology.

[70] Chen L.M., Zhang A.P., Wang F.F., Tan C.X., Gao Y., Huang C.X., Zhang Y., Jiang L., Zhou C.N., Chao F.L. (2016). Running exercise protects the capillaries in white matter in a rat model of depression. J. Comp. Neurol..

